# Case Report: hidradenocarcinoma presenting with lymph node metastasis: a diagnostic challenge and the pivotal role of morphology

**DOI:** 10.3389/fonc.2026.1810864

**Published:** 2026-04-01

**Authors:** Lun Li, Lidi Tian, Tao Wang

**Affiliations:** 1 Department of Dermatology, Ya’an People’s Hospital, Ya’an, China; 2 Department of Pathology, Ya’an People’s Hospital, Ya’an, China

**Keywords:** carcinoma of unknown primary, diagnostic delay, hidradenocarcinoma, lymph node metastasis, morphology

## Abstract

Hidradenocarcinoma (HAC) is a rare malignant sweat gland tumor. When presenting with lymph node metastasis and an occult primary lesion, diagnosis is often significantly delayed. We report a case of a 58-year-old man with right axillary lymphadenopathy in whom the primary tumor remained elusive despite extensive workup, including two lymph node biopsies, multiple immunohistochemical(IHC) panels, and a PET-CT scan. This led to an initial diagnosis of carcinoma of unknown primary (CUP) and the initiation of empirical chemotherapy. Ultimately, a crucial morphological clue—”features favoring cutaneous sweat gland origin”—was identified on a subsequent biopsy, which prompted a systematic dermatological examination. The subsequent dermatological workup yielded the primary lesion: an 8 × 10 cm hidradenocarcinoma on the back, which was deemed inoperable due to its advanced stage. This case highlights a common diagnostic blind spot for cutaneous origins in CUP and underscores the critical role of meticulous histomorphological analysis when immunohistochemistry is equivocal. We thus advocate for the mandatory inclusion of a comprehensive full-body skin examination in the standard diagnostic protocol for CUP to prevent delays in diagnosing such rare yet treatable skin cancers.

## Introduction

Hidradenocarcinoma is an exceedingly rare adnexal skin cancer, accounting for approximately 0.05% of all skin malignancies. It predominantly affects males (60%) and is typically diagnosed around the age of 60 (1). The diagnostic challenge escalates when the disease presents with metastatic lymphadenopathy without an evident primary skin tumor (2). In such scenarios, clinical and pathological efforts are invariably focused on more common visceral sources—such as breast, lung, or gastrointestinal primaries—while the possibility of a rare cutaneous origin is often overlooked (3). Consequently, patients are frequently misclassified as having carcinoma of unknown primary (CUP), undergoing suboptimal management and missing the window for potentially curative local therapy. We present a case of metastatic hidradenocarcinoma with a five-year diagnostic delay, highlighting the pivotal role of morphology and advocating for routine skin examination in CUP workup.

## Case report

A 58-year-old male presented in July 2025 with a five-year history of progressive right axillary lymph node enlargement. He first noticed a painless right axillary nodule in 2020. By October 2020, it had grown to 1.5 × 4 cm, prompting an excisional biopsy in February 2021. Histopathology: poorly differentiated metastatic carcinoma. IHC: CK7(+), CK5/6(+), P40(+), GATA-3 (focal+). TTF-1, CDX-2, PSA were negative. PET-CT (March 2021): No primary lesion found. Diagnosed with CUP, the patient underwent 6 cycles paclitaxel/carboplatin (Mar-Aug 2021), achieving stable disease.

**Figure 1:**
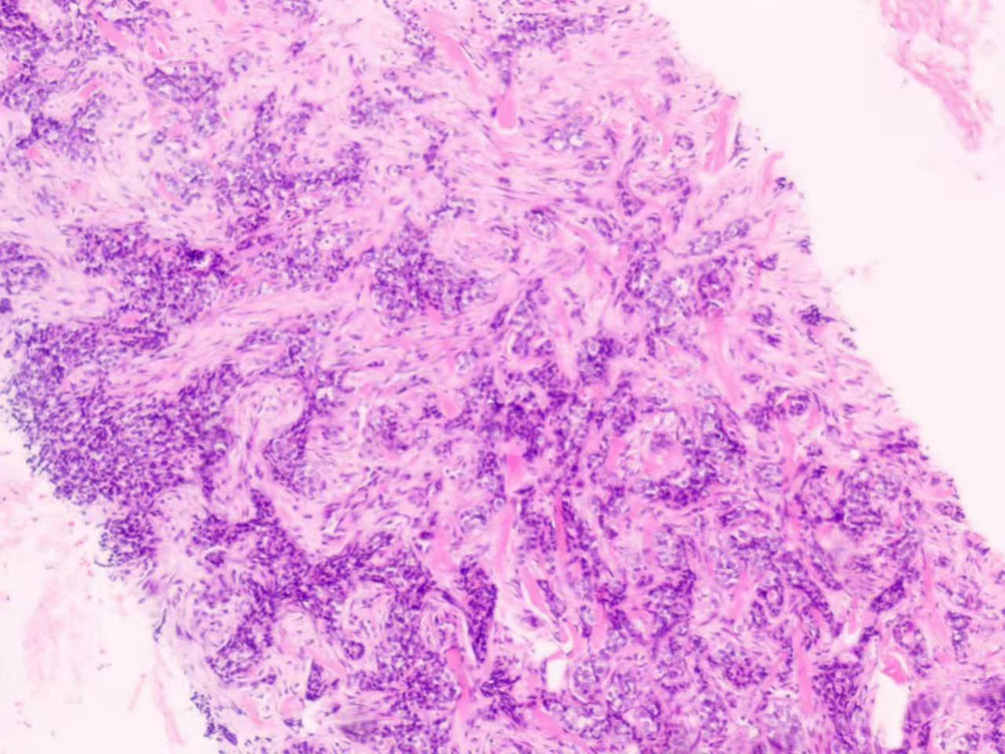
Lymph node metastasis: the morphological clue that changed the diagnosis H&E stain, ×100. Tumor cells infiltrate the lymph node, forming nests and irregular gland-like/tubular structures. This glandular architecture is a key morphological feature favoring a cutaneous sweat gland origin.

In July 2025, he presented with a new 2×2 cm right axillary node. Ultrasound-guided core biopsy revealed a nonspecific IHC profile (CK7+, CK5/6+, P40+, P63+, CKpan+, CK8/18+, CK19 partial+; Ki-67 ~25%), with negative markers for lung, breast, prostate, and gastrointestinal primaries. Representative IHC staining results for P63 and CK5/6 are shown in **Supplementary Figure 1**, **2**, respectively. On H&E-stained sections, the pathologist noted a key morphological feature: the tumor cells formed distinct nests and irregular gland-like structures. The pathology report explicitly stated: “Morphological features favoring cutaneous sweat gland origin” (**Figure 1**), prompting an urgent dermatology consult.

**Figure 2:**
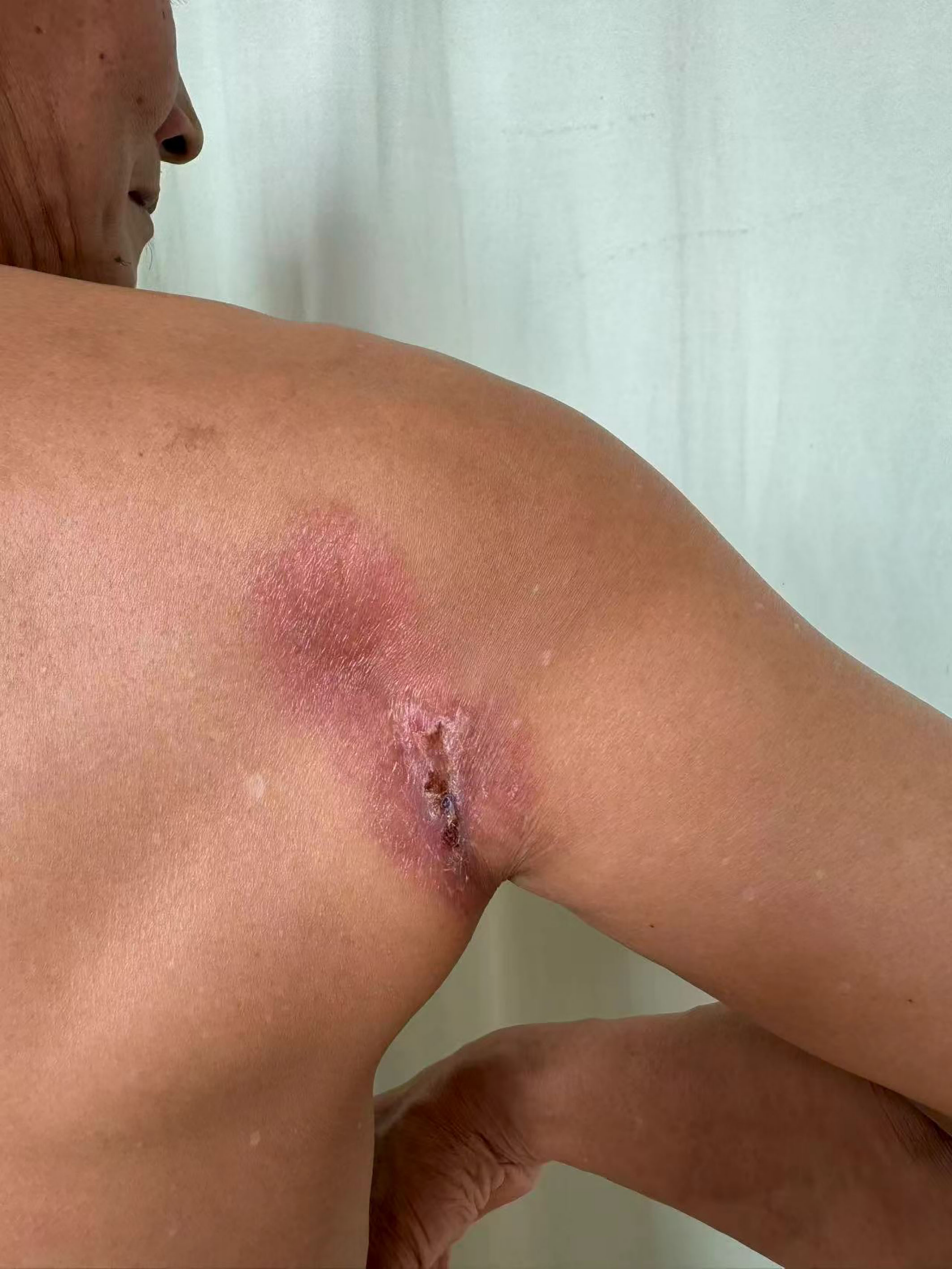
Clinical presentation of the primary lesion. An 8 × 10 cm infiltrative plaque on the right scapular region, featuring central ivory-white sclerotic atrophy and a dusky red infiltrative border. This indurated, long-standing lesion (present for years but overlooked during prior CUP workups) is clinically consistent with an invasive primary cutaneous carcinoma.

**Figure 3:**
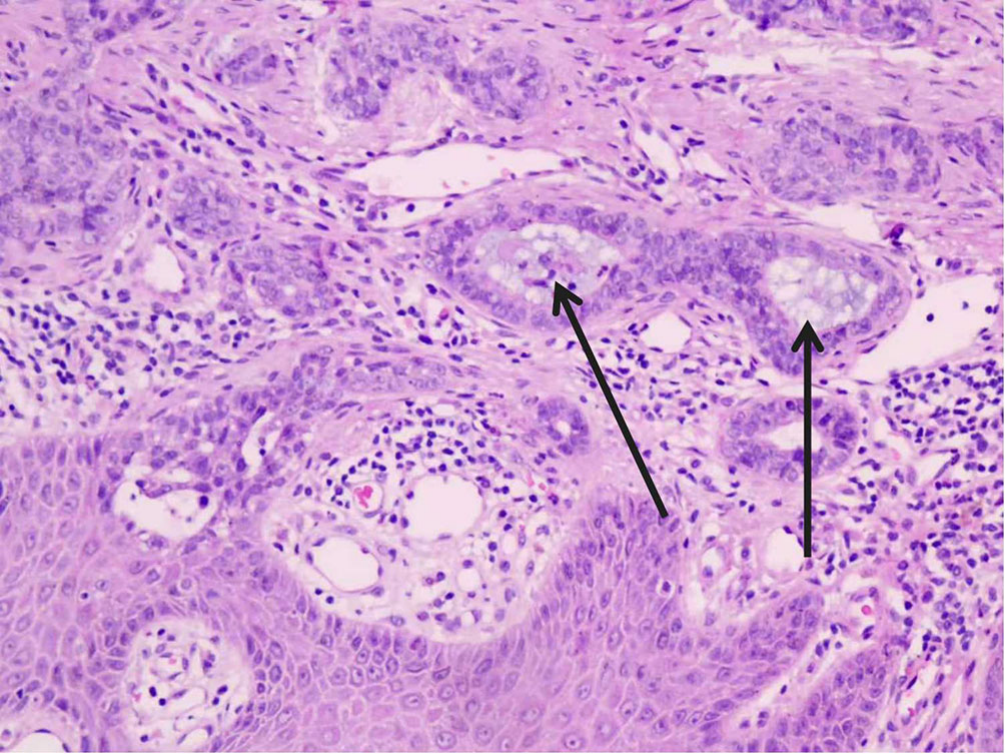
Histopathology of the primary tumor. H&E stain, ×200. The superficial dermis showed a diffuse proliferation of atypical glands and tubular structures. These exhibited focally cribriform and solid growth patterns. Cytologically, the tumor cells demonstrated significant nuclear enlargement and hyperchromasia. Intraluminal secretions were also noted (Shown by the arrow). The morphological features were consistent with a sweat gland origin.

Systematic full-body skin examination revealed an 8 × 10 cm indurated plaque on the right scapula, present for years (**Figure 2**). Dermoscopy showed arborizing telangiectasias (**Supplementary Figure 3**). Biopsy confirmed dermal infiltrative adenocarcinoma with glandular differentiation and desmoplastic stroma, diagnostic of hidradenocarcinoma (**Figure 3**; **Supplementary Figure 4**). The tumor was extensive (cT4N2M0, indicating locally advanced disease with regional lymph node involvement but no distant metastasis), precluding curative surgery. The patient was referred for palliative chemoradiotherapy. The diagnostic timeline is summarized in **Table 1**.

**Table 1:** Five-year diagnostic timeline from initial symptom to final diagnosis.

Date	Event	Key findings	Intervention	Outcome/significance
Early 2020	Patient self-detection	Painless right axillary nodule	None	Insidious onset
Oct 2020	First medical visit	Nodule 1.5 × 4 cm	Excisional lymph node biopsy	Metastatic carcinoma; non-diagnostic IHC
Mar 2021	Diagnostic workup	PET-CT: no primary lesion	Diagnosis of CUP	Diagnostic pathway diverted
Mar–Aug 2021	Empirical chemotherapy	Stable disease	Paclitaxel/carboplatin ×6 cycles	Partial disease control; primary lesion progresses
Aug 2021–Jun 2025	Follow-up	Clinically stable	Regular surveillance	Diagnostic stagnation
Jul 2025	Recurrence	New 2 × 2 cm axillary lymph node	Ultrasound-guided core needle biopsy	Diagnostic turning point
Jul 2025	Pathology review	H&E: glandular/tubular structures; “features favoring cutaneous sweat gland origin”	Urgent dermatology consultation	Diagnostic reorientation
Jul 2025	Dermatology examination	8 × 10 cm infiltrative plaque, right scapula	Biopsy of skin lesion	Final diagnosis: hidradenocarcinoma
Jul 2025	Staging	cT4N2M0, unresectable	Referred for palliative chemoradiotherapy	Clinical consequence of diagnostic delay

## Timeline

## Discussion

The persistent misdiagnosis of hidradenocarcinoma as carcinoma of unknown primary (CUP) underscores a critical blind spot in current diagnostic algorithms. Despite its aggressive potential, its often-indolent clinical presentation as a solitary, asymptomatic nodule (4)leads to frequent oversight until metastatic spread occurs. This pitfall is not unique to our case: a review of the literature reveals recurring patterns of misdiagnosis. Fortarezza et al. recently described two cases of hidradenocarcinoma with axillary lymph node metastasis, both initially misinterpreted clinically—one as a sebaceous cyst and another as an unspecified subcutaneous growth. Their systematic review identified 21 similar chest wall hidradenocarcinomas, highlighting how these tumors frequently mimic breast carcinoma (2). Even among other sweat gland neoplasms, diagnostic delays are well-documented: eccrine porocarcinoma has been reported to involve multi-year delays across multiple specialties (5). These cases collectively underscore a recurring theme—hidradenocarcinoma and related adnexal malignancies are frequently overlooked due to their indolent presentation, clinical mimicry of benign or more common conditions, and the tendency to attribute metastases to visceral primaries when the primary cutaneous lesion remains occult.

This case exemplifies a significant gap in the diagnostic algorithm for CUP, where cutaneous adnexal carcinomas remain frequently overlooked despite advances in diagnostic modalities. The reasons for this oversight are multifaceted. The initial focus on common visceral carcinomas, reinforced by non-specific immunohistochemistry (IHC) and negative PET-CT, created a “cognitive blind spot” for a cutaneous primary in this patient. Although IHC serves as a cornerstone for tumor classification, hidradenocarcinoma lacks a definitive immunoprofile and frequently shows overlapping features with other adenocarcinomas. Therefore, histopathological examination remains the gold standard for definitive diagnosis (6). In cases where IHC results are equivocal, morphological assessment of traditional H&E-stained sections often provides critical, decisive clues. In this case, the pathologist’s expertise was key to identifying characteristic infiltrative architectural patterns—such as glandular, ductal, and solid nests within the dermis—which provided the essential direction for the subsequent clinical investigation (7).

The diagnostic challenge is compounded by the fact that the differential diagnosis of hidradenocarcinoma includes several entities with overlapping histology. The most clinically relevant in the CUP context are squamous cell carcinoma (SCC), metastatic adenocarcinoma, squamoid eccrine ductal carcinoma (SEDC), and cutaneous metastases from visceral primaries. Squamous cell carcinoma may show nested growth similar to hidradenocarcinoma, but lacks true glandular structures and is typically negative for CEA and EMA (7). Metastatic adenocarcinoma (e.g., from lung or breast) shares glandular patterns, but retains organ-specific markers (TTF-1, GATA-3) and lacks the prominent intraluminal secretions characteristic of hidradenocarcinoma (3). In our case, PET-CT and CT scans excluded a visceral primary. Squamoid eccrine ductal carcinoma, a rare adnexal malignancy, contains ductal components reminiscent of hidradenocarcinoma, but its ductal differentiation is less prominent and secretory activity is typically sparse (8). Cutaneous metastases from visceral primaries (e.g., gastrointestinal, renal) may mimic primary adnexal neoplasms, but their morphology generally recapitulates the primary site, and they express lineage-specific markers such as CDX-2 or PAX8 (3, 6). These were also excluded by imaging in our patient. These overlapping morphological and immunohistochemical features explain why hidradenocarcinoma is frequently misdiagnosed, underscoring the critical need for thorough histopathological assessment with a comprehensive panel of markers and, crucially, correlation with clinical findings.

Beyond the pathological complexities, this case also highlights the consequences of omitting a systematic skin examination in the CUP workup. Primary hidradenocarcinoma lesions can be subtle, slow-growing, and asymptomatic, easily overlooked by patients and non-specialists. At our institution at the time of initial presentation, dermatologic consultation was not a mandatory component of CUP protocols. Importantly, the patient had previously consulted multiple healthcare facilities—including a tertiary referral hospital—and at none of them was a systematic skin examination performed during the CUP workup. This systematic gap, compounded by delays in communication between oncology, pathology, and dermatology, contributed to the five-year diagnostic odyssey. The delay in this case was not merely an individual oversight but reflects systemic deficiencies in current CUP diagnostic pathways: routine dermatologic evaluation is not yet embedded in standard protocols, and dermatologists are often not involved in the diagnostic process until cutaneous lesions become clinically evident.

The consequences of diagnostic delay are particularly significant given the aggressive nature of hidradenocarcinoma. For localized disease, surgery remains the mainstay of treatment—specifically, Mohs micrographic surgery and sentinel lymph node biopsy (9). Our patient ultimately lost the opportunity for curative surgery due to the prolonged diagnostic delay. However, advanced-stage hidradenocarcinoma lacks standardized therapy (1). Adjuvant radiotherapy may be considered for positive margins or unresectable disease, though its efficacy remains controversial. Evidence for systemic therapy is limited to case reports, with no established first-line regimen (1, 10). Various agents have been attempted—including 5-fluorouracil, capecitabine, platinum compounds, and doxorubicin—with variable responses. In our patient, six cycles of paclitaxel/carboplatin achieved lymph node regression and disease stability for over four years, aligning with sporadic reports of taxane-platinum efficacy in metastatic disease (11).

Targeted therapies have been reported in isolated cases—trastuzumab for HER2+ tumors (12), sunitinib in chemotherapy-refractory disease (13), and tamoxifen for ER+ tumors (10)—but none are currently approved for hidradenocarcinoma. Emerging data from the largest cohort to date (n=47) show that hidradenocarcinoma typically exhibits low PD-L1 expression and low tumor mutational burden, suggesting limited efficacy of immune checkpoint inhibitors (14).

However, robust population-based data on prognostic factors in metastatic hidradenocarcinoma remain scarce, and we acknowledge this as a limitation of the current literature.

## Conclusions

To prevent diagnostic failures in cases of suspected CUP, we propose the following actionable guidelines based on the lessons learned from this case:

Pathologists should provide descriptive morphological clues in their reports when IHC is indeterminate, explicitly suggesting the possibility of adnexal origin when architectural features (e.g., glandular, ductal, or nested growth with secretions) are observed.

Clinicians must include rare cutaneous malignancies in the differential diagnosis of CUP and conduct a systematic, full-body skin examination as part of the initial workup. Whenever feasible, this examination should be performed by a dermatologist to maximize sensitivity for subtle lesions.

Institutions should integrate mandatory dermatology consultation into standardized CUP diagnostic protocols. Furthermore, establishing structured multidisciplinary team (MDT) pathways involving oncology, pathology, and dermatology is essential. Routine MDT discussion for all CUP cases with non-diagnostic IHC or unusual patterns of metastasis can facilitate earlier recognition of rare primaries.

Recognizing the limits of technology and reaffirming the value of fundamental clinical and pathological skills remain crucial in managing such complex diagnostic challenges. Hidradenocarcinoma presenting with lymph node metastasis is a rare but important mimicker of CUP, and a high index of suspicion, combined with systematic multidisciplinary collaboration, offers the best chance for timely diagnosis and potentially curative intervention.

## Patient perspective

“For five years, I went from hospital to hospital, test to test—blood draws, CT scans, PET-CT, two surgeries. Every time, the doctor said, ‘We still can’t find the primary.’ I felt like I was walking in the dark, not knowing where the end was.

The truth is, I had a lump on my back the whole time. At first, it was just a small hard spot. It didn’t hurt, so I didn’t think about it. Over the years, it grew slowly, but I told myself it was just an age spot, a skin tag. I never mentioned it to any doctor. And not a single doctor ever asked me to take off my shirt.

When the pathologist said, ‘This might be from the skin,’ and the dermatologist finally examined my whole body—and found that palm-sized lesion on my back—I had so many mixed feelings. I blamed myself for not speaking up earlier. But I also couldn’t help wondering: why, in all those years, had no doctor ever asked me to undress?

Now I know my cancer is too advanced for surgery. I’m sharing my story so that doctors will remember: sometimes the primary is not hidden—it’s just overlooked. And so that other patients like me will know: any spot on your skin that doesn’t go away deserves to be seen by a doctor”.

## Data Availability

The original contributions presented in the study are included in the article/**Supplementary Material**. Further inquiries can be directed to the corresponding author.

## Supplementary material

Supplementary Figure 1 Immunohistochemical Staining for P63. Micrograph (magnification ×100) showing positive nuclear staining for P63 in the tumor cells within the lymph node metastasis. This marker was partially positive but non-specific for lineage determination.

Supplementary Figure 2 Immunohistochemical Staining for CK5/6. Micrograph (magnification ×100) demonstrating diffuse cytoplasmic positivity for CK5/6, confirming epithelial origin but lacking specificity for hidradenocarcinoma.

Supplementary Figure 3 Dermoscopic Features of the Primary Lesion. Polarized dermoscopy revealed coarse, tortuous arborizing telangiectasias within the plaque on the right scapula. While supportive, these findings are not diagnostic of hidradenocarcinoma.

Supplementary Figure 4 Additional Histopathology of the Primary Tumor. **(A)** Low-power view (H&E, ×100) showing the overall infiltrative architecture of the dermal tumor. **(B)** High-power detail (H&E, ×200) demonstrating intraluminal secretory material and marked nuclear pleomorphism.
