# Supplementary figures and images for: Case Report: hidradenocarcinoma presenting with lymph node metastasis: a diagnostic challenge and the pivotal role of morphology
Source: Front Oncol. 2026 Apr 1;16:1810864. doi: 10.3389/fonc.2026.1810864 (PMC13079154; doi:10.3389/fonc.2026.1810864)

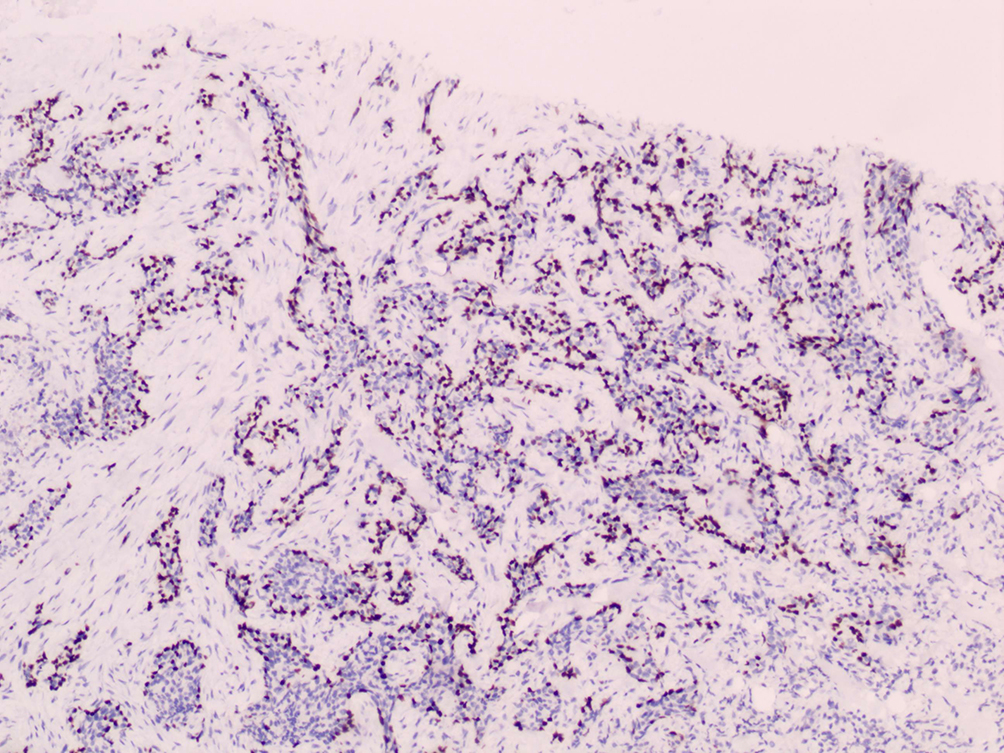

Supplement: Supplementary Figure 1 — Immunohistochemical Staining for P63. Micrograph (magnification ×100) showing positive nuclear staining for P63 in the tumor cells within the lymph node metastasis. This marker was partially positive but non-specific for lineage determination. [file Image1.jpeg]

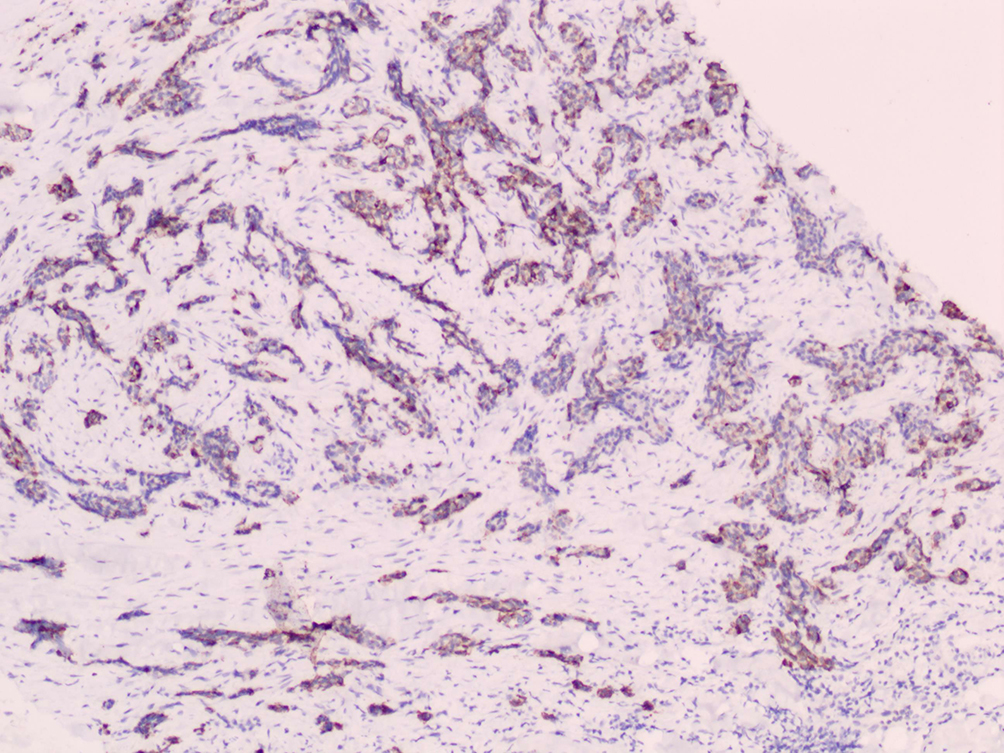

Supplement: Supplementary Figure 2 — Immunohistochemical Staining for CK5/6. Micrograph (magnification ×100) demonstrating diffuse cytoplasmic positivity for CK5/6, confirming epithelial origin but lacking specificity for hidradenocarcinoma. [file Image2.jpeg]

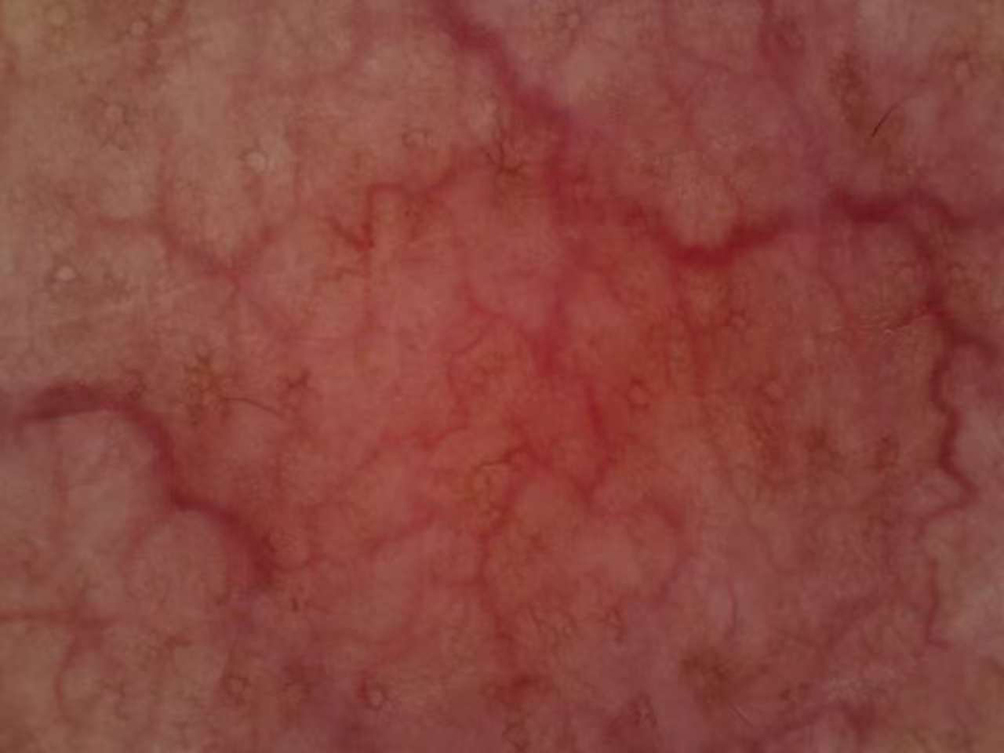

Supplement: Supplementary Figure 3 — Dermoscopic Features of the Primary Lesion. Polarized dermoscopy revealed coarse, tortuous arborizing telangiectasias within the plaque on the right scapula. While supportive, these findings are not diagnostic of hidradenocarcinoma. [file Image3.jpeg]

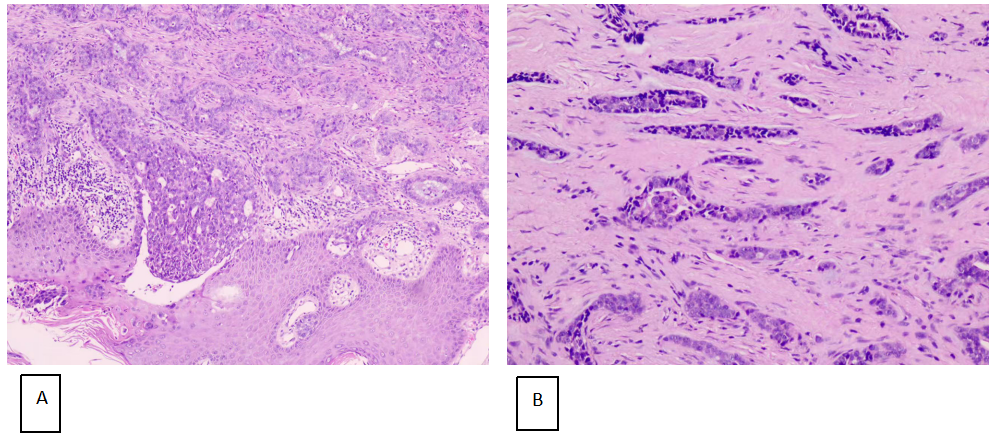

Supplement: Supplementary Figure 4 — Additional Histopathology of the Primary Tumor. (A) Low-power view (H&E, ×100) showing the overall infiltrative architecture of the dermal tumor. (B) High-power detail (H&E, ×200) demonstrating intraluminal secretory material and marked nuclear pleomorphism. [file Image4.png]
